# An Evaluation of Dental Caries Status in Children with Oral Clefts: A Cross-Sectional Study

**DOI:** 10.3390/ijerph22020227

**Published:** 2025-02-06

**Authors:** Lucimara Teixeira das Neves, Beatriz Costa, José Roberto Pereira Lauris, Ana Lídia Ciamponi, Marcia Ribeiro Gomide

**Affiliations:** 1 Department of Biological Sciences, Bauru School of Dentistry and Post-Graduation Program in Rehabilitation Sciences, Hospital for Rehabilitation of Craniofacial Anomalies, University of São Paulo (HRAC/USP), Bauru 17012-900, Brazil; 2 Department of Pediatric Dentistry, Hospital for Rehabilitation of Craniofacial Anomalies, University of São Paulo (HRAC/USP), Bauru 17012-900, Brazil; biacosta@usp.br (B.C.); marcinhargomide@gmail.com (M.R.G.); 3 Department of Pediatric Dentistry, Orthodontics and Public Health, Bauru Dental School, University of São Paulo, Bauru 17012-900, Brazil; jrlauris@usp.br; 4 Department of Pediatric Dentistry, São Paulo Dental School, University of São Paulo, São Paulo 05508-220, Brazil; analidia@usp.br

**Keywords:** dental caries, tooth, deciduous, oral cleft, nonsyndromic

## Abstract

Oral health is a prerequisite for the rehabilitation of children with oral cleft (OC). Thus, caries negatively affects individuals with OC. This study aimed to investigate dental caries in children with OC, determining the first period of the most significant susceptibility to caries. This cross-sectional study evaluated caries’ prevalence and mean dmft in the primary dentition of 300 children with OC, distributed among ten groups at six-month intervals. The exams were carried out using the WHO diagnostic criteria. Fisher’s exact test and Student’s *t*-test were used for statistical comparisons with a significant level of 5%. The caries prevalence in the total group was 59.4%, and the dmft was 3.4. The first period of susceptibility occurred between 13 and 18 months and 19 and 24 months, with a prevalence ranging from 6.6% to 40% (*p* < 0.05) and a dmft ranging from 0.1 to 1.0 (*p* < 0.05). The prevalence and dmft increase with age. Our findings suggest that in children with OC, the first period of susceptibility to caries occurs from 13 to 18 months. These findings demonstrate the need for pediatric dentistry to establish an early preventive protocol for children with clefts during the first year.

## 1. Introduction

Oral cleft (OC) is a common congenital craniofacial anomaly with a global prevalence of 0.15% per live birth, with an estimated 348,000 babies born with OC yearly [1,2]. This condition causes anatomical and dental alterations and requires long-term treatment by a multidisciplinary team [3,4,5]. Considering the type of OC, the rehabilitation process should start early, in the first year of life, with surgical repair [6]. In this context, the occurrence of caries negatively affects individuals with OC since oral health is a prerequisite for rehabilitation [7]. Dental caries is a multifactorial and progressive disease that involves the interaction between microbiological, dietary, and environmental factors, with a direct impact on dental health and the quality of life of individuals [8]. Caries is a public health problem and, as such, is a challenge to the good oral health of children with OC [9]. The study of caries experience in individuals with oral cleft (OC) is essential for outlining the vulnerable aspects of proper oral health because of its importance for rehabilitation, especially regarding reparative surgeries since caries or any other infection in the oral cavity might impair the success of these procedures [3,6,7,10].

The relationship between the oral cleft and caries prevalence has been investigated [1,3,11,12,13,14,15,16,17]. Some authors believe that more severe types of clefts increase caries prevalence [18,19]. On the other hand, other authors did not find any difference [15,16,17,20,21,22]. In general, the prevalence of caries in children and adolescents with OC, in most studies, is presented in a broad and general way without detailing the disease in periods. No study has investigated the caries experience, especially in primary dentition, considering short age intervals throughout early childhood and preschool. By analyzing this aspect, it is possible to understand in more detail the period of susceptibility in which there is an increase in the caries experience. This type of study is justified because knowing the caries experience profile in childhood has been considered a predictor for the occurrence of this pathology in adolescence and throughout life, affecting long-term oral health [23,24,25].

According to the understanding of the WHO, the investigation of the caries experience in different populations has as its primary objective to offer an overview of the oral health condition of a population with specific particularities, such as the population of children with oral clefts (OCs). Therefore, it is crucial to determine periods of greater susceptibility and highlight the need for prevention and intervention to control the disease. This detailed analysis of the disease status in children with OC is essential because it allows for establishing clinical practice guidelines with preventive strategies specifically targeted at this population [10,26]. Thus, this study aimed to investigate the dental caries status in the different stages of the primary dentition of children with OC and determine the first period of susceptibility to increased caries. The null hypothesis is that dental caries status does not increase with age in the deciduous dentition of children with oral clefts.

## 2. Materials and Methods

This retrospective cross-sectional study was conducted at the Hospital for Rehabilitation of Craniofacial Anomalies—University of São Paulo (HRAC-USP). According to the principles of the “Declaration of Helsinki”, it was approved by the Human Research Ethics Committee of the HRAC-USP. All parents of the children included in the study were given verbal explanations and asked to sign the informed consent form to participate in the study.

### 2.1. Study Participants

The sample size was calculated using the G*Power (version 3.1, Heinrich Hei-ne-Universität, Dusseldorf, Germany) program. Considering data from the literature on the prevalence of caries in children with OC in early childhood (20%—between 0 and 3 years) and at preschool age (71%) [5,27], it was determined that the number of samples adopting α error at 0.05 and β error at 0.10 (power = 0.90) should be a minimum of 18 in each age group.

A total of 300 children (150 boys and 150 girls) participated in this study. The screening process considered as inclusion criteria that the child should be between 7 and 66 months old and have been diagnosed with nonsyndromic cleft lip and alveolus (NSCLA) or nonsyndromic cleft lip and palate (NSCLP). Children with other types of clefts, associated anomalies, or syndromes were excluded. The recruited participants were homogeneously divided into ten groups with 30 children, each matched by sex (15 boys and 15 girls), with an age interval every six months (Table 1). According to the criteria described above, this recruitment and clinical examination occurred during the routine dates for children’s care at the hospital (HRAC-USP).

### 2.2. Clinical Examination

All children were examined for dental caries lying on a medical bed under natural and artificial light with the aid of a dental mirror and Community Periodontal Index Probe (CPI Probe) for removal of debris from the teeth. The examination was performed by a single calibrated examiner and experienced LTN. A pilot study was performed before the initiation of data collection to assess intrarater reliability using Cohen’s kappa score. For intrarater reliability, 30 random children enrolled at the HRAC-USP were examined at two different time points. The kappa coefficient was 0.98, which demonstrated intraobserver solid reliability.

For caries evaluation, the diagnostic criteria established by the World Health Organization (WHO) were employed [28]. All teeth that crossed the gum tissue, with some visible portions, were considered erupted. The status of the deciduous dentition was recorded using the dmft index (decayed, missing, and filled teeth index). The dmft index was calculated for each subject. The method used for the intraoral exam was systematic per quadrant. The sequence of examination was clockwise from the last maxillary right tooth to the last mandibular right tooth. No dental radiographs were taken.

Dental caries experience was assessed by prevalence, mean dmft, and distribution.

Prevalence was determined for the percentage of children with past or present caries experience in each age group. Caries experience (present or past) was considered when dmft ≠ 0 and without caries (caries-free) when dmft = 0. The mean number of teeth affected by caries in each group was indicated by the dmft index. Distribution was the observation of the frequency of occurrence of caries in each type of tooth.

After the assessment proposed in the study, the parents of all participants were advised on dietary habits and oral hygiene, and those children who had cavities were referred for treatment.

### 2.3. Statistical Analysis

Descriptive statistics (i.e., frequencies, percentages, means ± standard deviations (SDs)) were performed. Categorical variables were expressed as numbers and percentages (%). Univariate analyses used the Chi-square test and Fisher’s exact test to assess the differences in the prevalence between age groups, comparing the frequencies of the age groups sequentially. Student’s *t*-test was applied to analyze the differences in the mean dmft values between sexes and between age groups. A multiple linear regression analysis for the dmft caries score was performed, including the age group, sex, and cleft type, to analyze the significance of each of them as a predictive variable of caries in this evaluated group of children with OC. The statistical analysis was performed using R statistical software (version 4.0.2; R Foundation for Statistical Computing, Vienna, Austria). Statistical significance was set as *p* < 0.05.

## 3. Results

**Figure 1:**
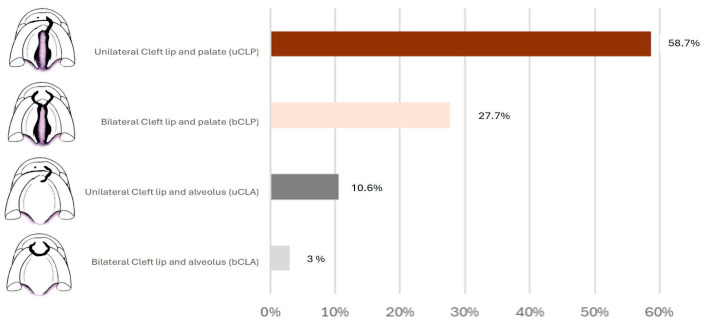
Sample distribution by cleft type.

The prevalence of caries in the total group was 59.4%, with a mean age of 36 months. The mean decayed, missing, and filled teeth (dmft) index was 3.4 ± 4.51 for the total group. There was no statistically significant difference between the sexes for the prevalence (61% for males and 58% for females) (*p* = 0.638) or the mean dmft index (3.54 for males and 3.27 for females) (*p* = 0.609). The cleft types of the sample are shown in Figure 1, where it is possible to observe a predominance of unilateral cleft lip and palate (*n* = 176).

Considering each age group and comparing the caries prevalence and the mean dmft between sexes in each age group, no statistically significant differences were found. Thus, data on caries experience were pooled between sexes into a single group to present the results.

**Figure 2:**
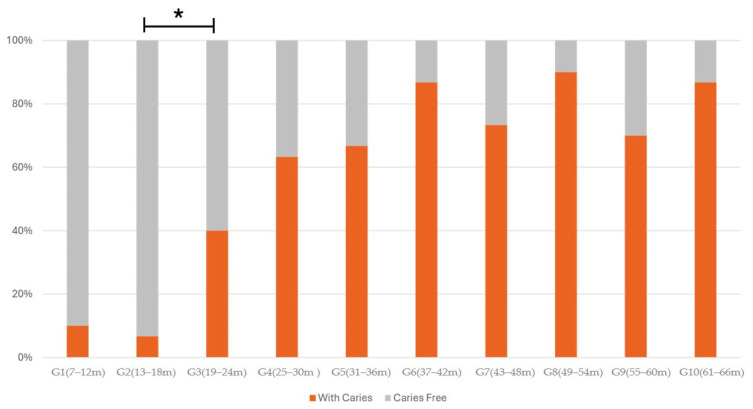
Caries prevalence for each age group. Comparison between G2 and G3 was statistically significant (* *p* = 0.005).

Caries prevalence was progressive, increasing with age, occurring in 10% of children aged 7–12 months and 86.6% of children aged 61–66 months (Figure 2). Regarding the period of increasing prevalence, the first statistically significant period occurred between 13 and 18 months and 19 and 24 months, with an increase in prevalence from 6.6% to 40%, respectively (*p* = 0.005).

**Figure 3:**
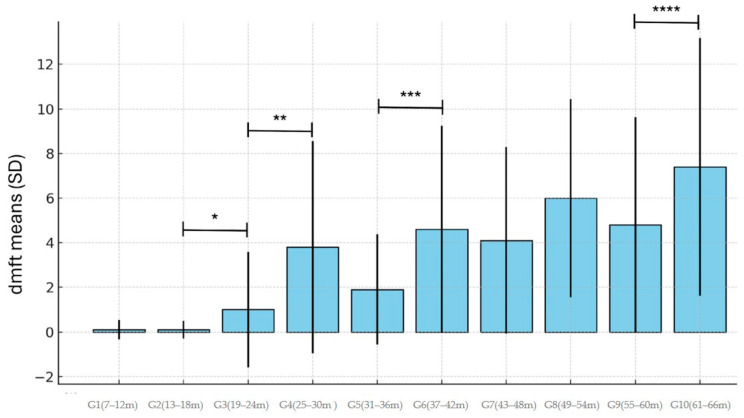
The mean dmft ± standard deviation for each age group. The following comparisons were statistically significant: G3 > G2 (* *p* = 0.02); G4 > G3 (** *p* = 0.003); G6 > G5 (*** *p* = 0.004); G10 > G9 (**** *p* = 0.03).

The mean of the dmft index also increased with age despite some variations between groups (Table 2 and Figure 3). The first period of the increase that was statistically significant also occurred from 13 to 18 months to 19 to 24 months, with values of 0.1 ± 0.40 and 1.0 ± 2.59, respectively (*p* = 0.02). Other statistically significant differences in the mean of dmft also occurred in other age groups, as shown in Figure 3.

A multiple linear regression analysis showed that of all factors included in the proposed model, only the age factor was significant (*p* < 0.001). This result revealed that the increasing age was significantly associated with increased dental caries (Table 3).

**Figure 4:**
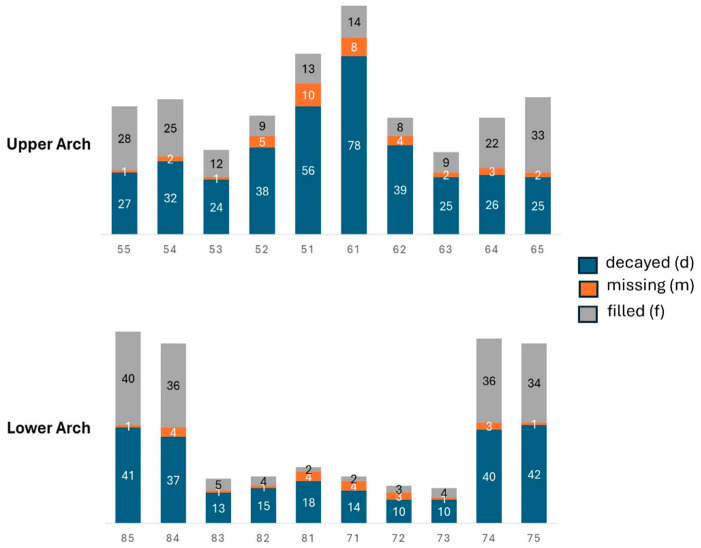
Total number of decayed (d), missing (m), and filled (f) teeth for each type of tooth and dental arch.

Of the 300 children evaluated, a total of 4813 teeth were examined. Of these, 1025 were computed in the dmft index. The maxilla was the most affected arch (*n* = 592). The distribution of caries for each type of tooth in the total group revealed higher caries experience in the central incisors of the maxilla. In the mandibular arch, the second and first molars were the teeth most affected (Figure 4). These results for the upper arch point to a possible correlation between the cleft and dental caries, especially in the upper anterior teeth adjacent to the area affected by the cleft.

## 4. Discussion

The null hypothesis was rejected. There was an increase in dental caries status in the deciduous dentition of children with oral clefts with increasing age.

At this point, the literature on caries in subjects with OC is still controversial. Some authors have reported the highest caries experience in subjects with OC compared to those in controls without clefts [1,3,11,12,13,14]. Other researchers could not observe this difference [15,16,17]. A recent literature review published by Grewcok and collaborators points out a few studies on the experience of caries, especially in primary dentition in children with NSOC [1]. Some limitations for comparison with other studies involve that some analyzed older children or vast age groups or even analyzed samples with different types of clefts [1,12,13,14,29]. Other studies present limitations for the comparison of the results, with too wide age ranges and the presentation of data divided according to the type of cleft, thus reducing the sample size, as well as differences in the methods employed for caries diagnoses [11,12,20,21,26]. Grewcock et al. [1] indicated that clinical studies investigating the dental caries in children with clefts can help in training future dentists in the clinical management of these children, as well as in allocating resources for public policies aimed at prevention in oral health for this population [1].

A differential of the present study is that a significant number of cases were analyzed, with standardized cases of cleft lip and alveolus or cleft lip and palate, and stratified by age group in a paired way in an early stage of childhood. This period in which hygiene and diet habits are established is directly related to the occurrence of caries throughout life. This selection criterion considering the type of cleft is essential since in cases where the clefts involve the alveolar ridge, dental anomalies in the region are more common, as well as greater difficulty or fear of parents in cleaning, predisposing them to caries due to poor oral hygiene [3,16,27,30].

An evaluation of the caries prevalence in the present study demonstrated a progressive increase with increasing age. The variation was 10% from earlier ages (7–12 months) to the highest prevalence (90%) in the 49–54-month age group. Notably, the occurrence of caries in this group with NSOC may be regarded as early since 10% of the children in the first age range evaluated (7–12 months) were affected, and the criterion for a caries diagnosis [28] considers the presence of caries when cavities may already be observed. A possible hypothesis for these findings is that the first reconstructive surgeries occur in this age group. Cheiloplasty, a surgery that repairs the lip, is ideally performed in the first year of life. With this surgery, scar tissue forms on the upper lip, making mouth hygiene difficult due to restricted lip mobility, which is quite common [16]. Parents generally report fear of manipulating the child’s mouth for dental hygiene. In addition, the early introduction of the bottle for feeding, due to the difficulties in natural breastfeeding, also can impact in the initial phase of primary dentition.

The first increase in caries prevalence occurred between 13 and 18 months (6.6%) and 19 and 24 months (40%). These prevalence findings are higher than those observed in a study conducted in Nigeria, which found no caries lesions in children with clefts aged 0 to 2 years [26]. Considering the other broader age groups in the preschool phase, our prevalence data are higher than those observed in other studies investigating children with clefts from other ethnic populations. Okoye and collaborators, evaluating Nigerian children aged 3 to 6 years, reported a 25% prevalence [26]. Likewise, Kirchberg and colleagues, who evaluated German children aged 1 to 6 years, reported a caries prevalence of 25% [31]. On the other hand, prevalence rates close to those found in the present study were observed by Briton and colleagues studying the age group from 4.5 to 6 years old at a prevalence of 68.2% [32]. In children with an average age of 57 months, Chopra and colleagues reported a prevalence of 71.9% [27].

Similarly to the increase in prevalence, the first increase in the mean dmft occurred from 13 to 18 months to 19 to 24 months. The variation in mean dmft for all age ranges was not uniform; instead, it was mediated by lower values in some groups, with a general variation from a minimum of 0.10 (at 13–18 months) to a maximum value of 7.4 (at 61–66 months). Briton and collaborators (2010), who evaluated age groups at 1-year intervals, reported a mean dmft of 0.49 in the range of 1.5 to 2.5 years, 1.03 between 2.5 and 3.5 years, and 0.94 between 3.5 and 4.5 years [32]. The present study found dmft values of 2.4, 3.28, and 5.05 in these same age ranges, respectively. A comparison of these findings revealed that the dmft values in the present study were, on average, 3 to 5 times higher. Okoye et al. reported a mean dmft of 0.38 in children between 3 and 6 years old [26]. In the present study, the mean dmft for similar age groups was 5.38, 14 times higher than those reported by these authors.

At isolated ages, the mean dmft observed in this study (from 6.0 at 49–54 months to 7.4 at 61–66 months) was higher than the results obtained in other studies, indicating that the dmft ranged between 1.3 and 3.8 [17,19,27,32].

Despite the different results found in these studies, it is important to highlight that the studies were conducted with children with other types of clefts and, in addition, represent different socioeconomic achievements, which may impact their oral health conditions. Nevertheless, they corroborate a tendency for the experience of caries to increase with age in early childhood and preschool.

An evaluation of the caries distribution in the primary dentition revealed that the maxillary central incisors were most affected, followed by the lower molars. These results corroborate the findings of other authors who also found a higher occurrence of caries in central incisors and lower molars [11,26,33].

A possible explanation for these findings is that, in addition to the presence of the cleft, the anterior teeth, especially in the cleft area, are susceptible to tooth anomalies of shape, number, position, and structure, which predisposes them to caries [3,4,15,16,18,20,34]. In addition, the scarring resulting from plastic lip rehabilitation surgery can make oral hygiene difficult in this area. The psychological aspects of caregivers who fear performing oral hygiene in the cleft area should also be considered [3,35]. The occurrence of tooth decay in the anterior region has several impacts on the child. Eating and speech are impaired, as well as psychological and social aspects, which are added to the presence of the cleft itself, which brings numerous stigmas.

In summary, in our study, we found that, in Brazilian children with NSOC, the first period that increased the prevalence and mean dmft was between the ages of 13 and 18 months and 19 and 24 months. It is essential to highlight that the prevalence rate indicates the number of children affected without considering the number of teeth involved, measured by the dmft index. In the present study, both indices increased during the same age transition, which means that at the same time, we observed a more significant number of children with cavities and a greater number of teeth affected by the disease in these children. These findings can involve several possible related general and specific factors.

As a general factor, the mechanism of caries disease involves a multifactorial etiology in its development and progression. Thus, it involves intrinsic factors related to the characteristics of the host, such as salivary parameters, active contamination by cariogenic pathogens, and the immune response mechanism against bacterial infection. It also involves extrinsic factors related to risk habits and behavior, such as diet and oral hygiene [21,24,36].

Specific factors in children with clefts include local anatomical particularities due to local characteristics such as the presence of the cleft itself, surgical repair, and scarring resulting from the surgery, which leads to less mobility of the upper lip. Regarding this age range specifically, our findings coincide with the postsurgical period of cheiloplasty and palatoplasty, which, in our surgical protocol at the hospital, occurs from 12 months onwards. Furthermore, this stage of development is an active phase of the eruption of deciduous teeth, with the eruption period of the first deciduous molars. The anatomy of these teeth and the frequent occurrence of dental anomalies lead to malalignment of the teeth, which further facilitates the accumulation of dental plaque, increasing the retentive areas and the number of microorganisms in the local microbiota [5,37,38]. In this phase, new foods are introduced into the diet, often with a preference for sugary foods. Moreover, from a developmental point of view, children are more resistant to parental help in brushing. However, they still do not have the fine motor coordination to perform oral hygiene efficiently, especially for the posterior teeth [38].

Furthermore, there were biological factors due to the various types of dental anomalies prevalent in children with clefts, such as supernumerary teeth, hypoplasia, and malocclusion, representing an additional risk factor for caries. Moreover, in addition to these anatomical and biological factors, there are also social and behavioral aspects, since it comprises children with low socioeconomic backgrounds, and limited access to dental care, hygiene products, and preventive information/reassurance, in addition to sociocultural variables related to sugar intake, as well as psychological aspects that intensify this risk status [10,16,38]. In some instances, this gives rise to parents’ permissible reactions regarding dietary and oral hygiene habits, among others [36,39]. In addition, for parents, the central aspect of rehabilitation is reconstructive plastic surgery, with less interest in the importance of oral health, which can lead to poor oral hygiene, increasing the risk of caries experience [12,27]. In this sense, some authors indicate early preventive care in oral health care for children with clefts [3,10,12,16,39].

This study had some limitations. The sample came from various regions of the country, and no data were collected on the family’s socioeconomic level or parents’ education level. Furthermore, covariate data, such as information on diet and oral hygiene habits, were not collected. A recommendation for future studies involving this type of investigation would be the distribution of the casuistry in short periods for the age group and the stratification of the sample for the different types of clefts. In addition to collecting information on hygiene and diet habits, complementing the risk estimation analysis allows for a better visualization of the different needs concerning the age groups and different types of clefts.

## 5. Conclusions

In conclusion, our results show that in the primary dentition of children with nonsyndromic oral clefts, the first period of susceptibility occurs from the age group of 13 to 18 months. And the mean dmft is higher in children in the older age group. These findings demonstrate the need and recommendation for the establishment, in the age group of 6–12 months, of an early preventive protocol of care to modify the way of thinking related to the emphasis on dental care and highlight the importance of teeth and good oral health conditions for the entire rehabilitative process and health promotion.

## Figures and Tables

**Table 1:** Distribution of study participants according to age and sex.

Age Groups (Months)	Male	Female	Total Number (%)
G1 (7–12 months)	15	15	30 (10%)
G2 (13–18 months)	15	15	30 (10%)
G3 (19–24 months)	15	15	30 (10%)
G4 (25–30 months)	15	15	30 (10%)
G5 (31–36 months)	15	15	30 (10%)
G6 (37–42 months)	15	15	30 (10%)
G7 (43–48 months)	15	15	30 (10%)
G8 (49–54 months)	15	15	30 (10%)
G9 (55–60 months)	15	15	30 (10%)
G10 (61–66 months)	15	15	30 (10%)
Total	150	150	300 (100%)

**Table 2:** The mean dmft (±standard deviation) and mean number of teeth for each age group.

Age Groups (Months)	Mean dmft ± Standard Deviation (SD)	Mean Number of Present Teeth
G1 (7–12 months)	0.1 ± 0.43	4.6
G2 (13–18 months)	0.1 ± 0.40	8.1
G3 (19–24 months)	1.0 ± 2.59	14.1
G4 (25–30 months)	3.8 ± 4.76	17.2
G5 (31–36 months)	1.9 ± 2.48	18.7
G6 (37–42 months)	4.6 ± 4.64	19.7
G7 (43–48 months)	4.1 ± 4.18	19.7
G8 (49–54 months)	6.0 ± 4.45	19.6
G9 (55–60 months)	4.8 ± 4.82	19.3
G10 (61–66 months)	7.4 ± 5.78	19.2
Total	3.4 ± 4.51	16.0

**Table 3:** Multiple Linear Regression for dmft score: Full Model including all variables.

Variable	Regression Coefficient	Standard Error	*p* Value
Intercept	−1.917	0.8671	
Age	0.131	0.0132	<0.001
Child Sex: Male–Female (Reference = F)	−0.388	−0.857	0.392
Type of Cleft (Reference = uCL)			
uCLP vs. uCLA	1.016	0.7512	1.352
bCLP vs. uCLA	0.634	0.8139	0.437
bCLA vs. uCLA	−1.850	1.4745	0.210

In this model, *R*^2^ = 26.3%. Legend: M, Male; F, Female; uCLP, Unilateral Cleft Lip and Palate; bCLP, Bilateral Cleft Lip and Palate; uCLA, Unilateral Cleft and Alveolus; bCLA, Bilateral Cleft Lip and Alveolus.

## Data Availability

The dataset generated and/or analyzed during the study is available from the corresponding author on reasonable request.
